# TGF-β cascade regulation by PPP1 and its interactors –impact on prostate cancer development and therapy

**DOI:** 10.1111/jcmm.12266

**Published:** 2014-03-15

**Authors:** Luís Korrodi-Gregório, Joana Vieira Silva, Luís Santos-Sousa, Maria João Freitas, Juliana Felgueiras, Margarida Fardilha

**Affiliations:** a Signal Transduction Laboratory, Centre for Cell Biology, Biology Department, Health Sciences Department, University of AveiroAveiro, Portugal

**Keywords:** TGF-β, PPP1, PIP, prostate cancer, phosphatase, signal transduction therapy

## Abstract

Protein phosphorylation is a key mechanism by which normal and cancer cells regulate their main transduction pathways. Protein kinases and phosphatases are precisely orchestrated to achieve the (de)phosphorylation of candidate proteins. Indeed, cellular health is dependent on the fine-tune of phosphorylation systems, which when deregulated lead to cancer. Transforming growth factor beta (TGF-β) pathway involvement in the genesis of prostate cancer has long been established. Many of its members were shown to be hypo- or hyperphosphorylated during the process of malignancy. A major phosphatase that is responsible for the vast majority of the serine/threonine dephosphorylation is the phosphoprotein phosphatase 1 (PPP1). PPP1 has been associated with the dephosphorylation of several proteins involved in the TGF-β cascade. This review will discuss the role of PPP1 in the regulation of several TGF-β signalling members and how the subversion of this pathway is related to prostate cancer development. Furthermore, current challenges on the protein phosphatases field as new targets to cancer therapy will be addressed.

## Introduction

### TGF-β signalling pathway—an overview

The transforming growth factor beta (TGF-β) superfamily comprises over 42 members, all of which are generated from a single pre-pro-peptide precursor. Besides, TGF-β1, 2 and 3, this superfamily includes the bone morphogenetic proteins (BMPs), the activins, the growth differentiation factors (GDFs) and the anti-muellerian hormone, among others 1. Virtually, all types of cells produce and are sensitive to TGF-β superfamily members. These play fundamental roles in several cellular processes such as cell proliferation, adhesion, differentiation, apoptosis and migration, which may vary according to the ligand, the tissue and the conditions 2.

Transforming growth factor beta is a cytokine, with pleiotropic effects, that is produced mainly by fibroblasts and epithelial cells 3. In the epithelium, TGF-β inhibits cellular proliferation 4, whereas in the mesenchyme, it promotes cellular proliferation 5,6. Other functions attributed to TGF-β are as follows: synthesis of extracellular matrix (ECM) 7, expression of integrins 8, modulation of immune response 9, angiogenesis 10 and wound healing 11. Bone morphogenetic proteins, on the other hand, display a broad range of effects, even though sharing similar structure and signal transduction mechanisms. Among these, bone and cartilage formation and embryogenesis are the most relevant 12,13. Activins play crucial roles in the activation of follicle-stimulating hormone 14, erythropoiesis 15 and survival of neurons 16.

Transforming growth factor beta family ligands dimerize, most commonly forming homodimers, and propagate the signal by interacting with membrane surface receptors presented in the target cell 17. A total of 12 transmembrane serine/threonine kinase receptors have been identified that are usually divided into two types: five constitutively active type II receptors (TGF-βRII) and seven non-constitutively active type I receptors (TGF-βRI). Co-receptors, which lack catalytic activity, have also been identified, namely endoglin (CD105) and betaglycan (TGF-βRIII) 17. Ligands display more affinity to the type II receptors, and the binding of the TGF-β to the type II receptor enables it to phosphorylate the GS domain of the type I receptor, activating its catalytic activity 2,18,19. The type I receptors are denominated activin receptors–like kinases (ALKs) and once activated exert their catalytic activity by phosphorylating the C-terminal SxS domain of the main intracellular signal transducers of the pathway, the Smads 20. Eight Smads have been identified in the human and mouse genomes: five regulatory Smads (R-Smads 1/2/3/5/8), one common Smad (Smad4, also known as Co-Smad) and two inhibitory Smads (I-Smads 6/7). The R-Smads, after being phosphorylated by the type I receptors, form trimers with the Co-Smad 20. In general, ALKs 1/2/3/6 propagate the signals *via* Smads 1/5/8, whereas ALKs 4/5/7 propagate it through Smads 2/3 2. The fine dynamic equilibrium between these two opposing pathways often determines the ultimate outcome of the signal. After the formation of the complex, it is then translocated to the nucleus *via* microtubules and dyneins 21,22. The nuclear import is mainly done by importins, although direct interaction with nucleoporins is also described 23. Once in the nucleus, the trimers act as trans-regulatory elements to activate or repress the expression of genes such as *Sp1*,*Id1* and *Myc*. R-Smads/Co-Smads complex can also recruit transcription co-activators or co-repressors to modulate the amplitude of the activation/repression of the transcription 2.

Moreover, besides activating Smad-dependent signalling, TGF-β can also activate other signalling pathways in a Smad-independent manner, such as mitogen-activating protein kinases (MAPKs), phosphatidylinositol 3-kinase (PI3K)/Akt and small GTPases 24,25.

### TGF-β in cancer

Transforming growth factor beta plays a pivotal role in a wide variety of diseases, as expected by its pleiotropic effects and ubiquitous expression, namely cardiovascular, connective tissue and neurological diseases, reproductive, developmental, skeletal and muscle disorders 26 and also in several cancers 27–32.

During cell malignant transformation, a number of alterations occur at molecular and cellular levels (genetic, epigenetic and somatic) and in the surrounding microenvironment, contributing to an increased survivability and proliferative advantage 27,33–35. The traditional hallmarks of cancer include: (*i*) insensitivity to anti-growth signals; (*ii*) evasion of apoptosis; (*iii*) self-sufficiency in growth signals; (*iv*) sustained angiogenesis; (*v*) limitless replicative potential; and (*vi*) tissue invasion and metastasis, all of which cooperate in providing malignant cells a selective advantage 36,37. Moreover, two new emerging hallmarks namely deregulation of the cellular energetics and avoidance of immune destruction have arisen 38.

In normal cells, a complex web of interconnected signalling pathways heavily regulates each of these functions. As a potent pleiotropic cytokine, TGF-β acts in normal tissues as a formidable barrier to the development of cancer hallmarks, 31 inhibiting cellular proliferation 39, migration and invasion 40, and promoting apoptosis 41, cell adhesion 40 and cellular differentiation 42. However, TGF-β plays a dual role in cancer, since in late-stage tumours, the cellular machinery subverts the signalling pathway to promote the cancer progression 29.

#### Insensitivity to anti-growth signals

**Figure 1:**
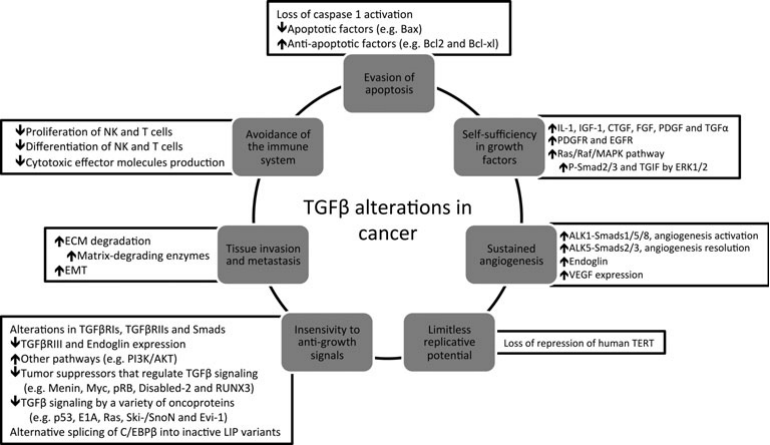
Alterations in TGF-β signalling cascade associated with major cancer hallmarks. The traditional cancer hallmarks are disrupted at variable extent, with insensitivity to anti-growth signals comprising the most well-described alterations. White boxes: TGF-β superfamily ligands, receptors, downstream effectors or in the responses exerted by TGF-β signalling pathway; Grey boxes: major cancer hallmark. TGF-β, transforming growth factor beta; IL-1, interleucin-1; IGF-1, insulin-like growth factor-1; CTGF, connective tissue growth factor; FGF, fibroblast growth factor; PDGF, platelet-derived growth factor; TGF-α, transforming growth factor alpha; PDGFR, PDGF receptor; EGFR, epidermal growth factor receptor; MAPK, mitogen-activated protein kinase; TGIF, transforming growth-interacting factor; VEGF, vascular endothelial growth factor; TERT, telomerase reverse transcriptase; pRb, protein retinoblastoma; LIP, liver-enriched inhibitory protein; ECM, extracellular matrix; EMT, epithelial–mesenchymal transition.

Transforming growth factor beta was initially named for its ability to promote the proliferation and transformation of mesenchymal cells in soft agar 43. However, it is now established that TGF-β inhibits epithelial, endothelial and hematopoietic cell proliferation 44. Transforming growth factor beta ability to mediate cytostasis occurs in the late G1 phase by preventing the progression to S phase through two synchronized events: (*i*) repression of *Myc*,*Id1* and *Id2;* and (*ii*) induction of cyclin-dependent kinase (CDKs) inhibitors p15^INK4b^, p21^CIP^ and p27^KIP^ 27,31,44,45. Nevertheless, virtually all epithelial-derived tumours (>85% of all human cancers) display partial or total resistance to TGF-β growth inhibition effects, being this a major cancer hallmark 27,31. Several mechanisms to override the cytostatic activities of TGF-β have been detected in cancer cells either due to inactivating mutations or repression of one or more genes of the TGF-β signalling pathway 32 (Fig. 1). As a result of this insensitiveness, cancer cells usually secrete larger amounts of TGF-β, being this production parallel to the cancer progression 28. Of possible relevance, it has been shown that TGF-βRI is sufficient to mediate some cellular responses to TGF-β, such as expression of *JunB* and *PAI-1*, indicating that maybe the receptor levels are sufficient to mediate the response *via* TGF-βRI, but not the ones *via* TGF-βRII 46,47.

#### Evasion of apoptosis

In general, TGF-β is pro-apoptotic in a Smad-dependent pathway 27,48, although Smad-independent pathways can also play a role 49. Apoptosis triggered by TGF-β can be both p53-dependent and p53-independent 27,50,51. In fact, p53 has been shown to interact with Smad2 in a TGF-β-dependent fashion, thus linking two of the most important apoptotic mediators in human cells 52. However, during malignant transformation, TGF-β signalling is altered to promote cell survival 31 (Fig. 1).

#### Self-sufficiency in growth signals

Cancer cells often acquire the ability to undergo proliferation when stimulated by TGF-β through the ability of inducing the expression of cytokines, growth factors and/or their receptors (Fig. 1). It has been also shown to activate the Ras/Raf/MAPK pathway 27. Therefore, by overexpression of growth signals or constitutive activation of downstream pathways, independence of exogenous growth factors is achieved 27.

#### Sustained angiogenesis

Angiogenesis is a normal physiological process in which new blood vessels are formed from pre-existing vessels, being essential in wound healing and embryogenesis. Solid tumours usually cannot exceed 1–2 mm because of limited access to oxygen and nutrients, being neoangiogenesis a way to circumvent this limitation. Ultimately, angiogenesis provides also a route for metastatic spread. *In vitro*, TGF-β can display either pro- or anti-angiogenic features. However, *in vivo* TGF-β is considered to have a pro-angiogenic role, being this action rather complex, as it appears to contribute to both phases of the angiogenic process: activation and resolution. Activation of ALK1 stimulates Smads1/5/8, regulating angiogenesis activation, while ALK5 regulates angiogenesis resolution *via* Smads2/3 27,31. Another relevant aspect is the fact that TGF-β induces the expression of VEGF in a Smad2/3 and Src-dependent mechanism, which directly contributes to angiogenesis 53. In cancer, pro-angiogenic effects of TGF-β seem to be up-regulated (Fig. 1).

#### Tissue invasion and metastasis

Transforming growth factor beta is a potent regulator of cell adhesion, ECM and motility. In normal cells, TGF-β stimulates the production of ECM by increasing the synthesis of collagen, fibronectin and other ECM proteins, decreasing the production of enzymes that degrade the ECM (*e.g*. heparinase, collagenase and stromelysin) and stimulating the production of proteins that inhibit ECM degradation (*e.g*. PAI-1 and TIM) (Fig. 1). However, cancer cells often respond to TGF-β by stimulating the expression of matrix-degrading enzymes, thus contributing to ECM degradation and invasiveness through epithelial-to-mesenchymal transition (EMT) 27. Epithelial-to-mesenchymal transition is a process that occurs during embryogenesis and a pathological feature in neoplasia, rheumatoid arthritis, chronic inflammation and fibrosis 29,31, which consists in the transdifferentiation of immotile, adherent, polarized epithelial cells into highly motile, apolar mesenchymal cells. Transforming growth factor beta has been found to promote EMT through a combination of Smad-dependent transcriptional events and Smad-independent effects on cell junction complexes 29 (Fig. 1).

#### Avoidance of the immune system

It has already been established that cancer initiation, promotion and progression are linked to aberrant and/or persistent inflammation within tumour microenvironment. In general, high levels of TGF-β are seen in advanced cancers and are found to inactivate host anti-tumour immunosurveillance systems, which confer immune privilege to developing neoplasms and ensures for their continued progression (Fig. 1). Transforming growth factor beta signalling crucial role has been demonstrated by the fact that Smad3-defficient mice exhibited defects in the responsiveness and chemotaxis of B and T cells and neutrophils 28,31. Therefore, it is now clear that TGF-β signalling affects all the hallmarks of cancer at some extent (Fig. 1). Cancer cells appear to selectively use the TGF-β responses that are advantageous.

### Phosphatases in cancer

A common mechanism used by cells to either propagate or terminate intracellular signal transduction pathways is the reversible protein phosphorylation 54. Many cellular processes are controlled by phosphorylation/dephosphorylation of structural or regulatory proteins that work as a molecular ‘switch’ 55,56. The reversible phosphorylation consists in the addition or removal of a negatively charged phosphate group mainly to serine, threonine or tyrosine residues calalyzed by protein kinases and protein phosphatases, respectively.

In humans, there are around 500 kinases, all sharing a related catalytic domain 57. Uncontrolled kinase activity is associated with cell proliferation and is a common finding in human cancers 58. Curiously, there are three to five times fewer phosphatases than kinases suggesting that the specificity of substrates is not only because of the catalytic subunits but also because of the regulatory subunits diversity 59.

Phosphoprotein phosphatase 1 (PPP1) is a major serine/threonine phosphatase and is expressed in all eukaryotic cells. This holoenzyme consists of a catalytic and at least one regulatory subunit. The catalytic subunit is encoded by three different genes (*PPP1CA*,*PPP1CB* and *PPP1CC*) that share near 90% of the amino acid sequence 55,60. The regulatory subunits account for PPP1 substrate diversity and consequently function 61,62. The regulatory subunits are known as PPP1 interacting proteins (PIPs) and, to date, more than 200 PIPs were identified with many more expected to be found 55,63. PIPs can be activity modulating proteins when their function is to inhibit or enhance PPP1 catalytic activity; targeting proteins, which are responsible for PPP1 subcellular localization or/and bring together PPP1 and specific substrates; and PPP1 substrates that associate with PPP1 and are dephosphorylated. Despite the function, every PIP interacts with PPP1. Although several PPP1 binding motifs have been already identified, such as RVxF and SILK, binding specificity between PIPs and PPP1 is obtained by differences in the number and the type of docking sites 64–67.

The evidence that reversible protein phosphorylation is essential for cellular function arises from the significant number of human diseases in which control of protein phosphorylation is impaired, like cancer and diabetes 60. In cancer, imbalances of protein phosphorylation appear to be an important pathophysiological mechanism 60. Constitutive activation of oncogenic kinases is one of the hallmarks observed in cancer cells, driving uncontrolled cell proliferation, invasion and metastasis 68. Transmembrane kinases, such as epidermal growth factor receptor and platelet-derived growth factor receptor, and cytoplasmic kinases, such as Raf and Akt, are mutated or hyperactivated in several types of human cancer. For example, an increased Akt signalling, a serine/threonine kinase involved in the control of cell size/growth, proliferation and survival 69, has been associated with poor clinical outcome in a variety of tumours, such as melanoma, breast and prostate 70.

Given that phosphorylation represents an essential element of cancer pathophysiology, it is not surprising that tumour suppressive functions have been linked to protein phosphatases 68,71,72. PPP2 and PPP1 have been associated with cancer suppressive processes, such as inhibition of cell survival, proliferation and migration 68,71. Moreover, PPP1 tumour suppressive functions have been connected with some of its PIPs 73.

## TGF-β signalling: role in prostate cancer pathogenesis

The different constituents of the prostate work as a functional unit, with the interactions between stroma and epithelium playing a pivotal role in normal prostate growth, development and function 74. It has been established that androgens, testosterone and dihydrotestosterone (DHT) are the most potent and relevant mitogens of the normal prostate 74. However, androgens actions are mainly indirect, through the stimulation of the production of diverse growth factors (*e.g*. EGF, TGF-α, KGF, IGF and bFGF) by stromal cells, which act mainly in a paracrine mode 74. To counteract the effects of these growth factors, both in stroma (*e.g*. FGF) and epithelium (*e.g*. TGF-α and EGF), TGF-β has been identified as a key growth modulator in normal prostate, by inducing growth inhibition and differentiation 27,47,74. Most prostate cancers arise as androgen-dependent, meaning that androgens drive cell proliferation. Prostate cancer progression usually involves the shifting to an androgen-independent state, sometimes with mutation or loss of the androgen receptor (AR) and an increasing impact of growth factors signalling pathways 75,76. This androgen resistance leads to an increase in the TGF-β production, which in turn promotes prostate cancer growth, viability and aggressiveness 47,77. Also, *in vitro* studies have shown that normal TGF-β-induced growth inhibition must be disrupted by the neoplasic surrounding environment because normal epithelial prostate cells are not able to protect themselves under any experimental condition 47.

### Ligands

**Figure 2:**
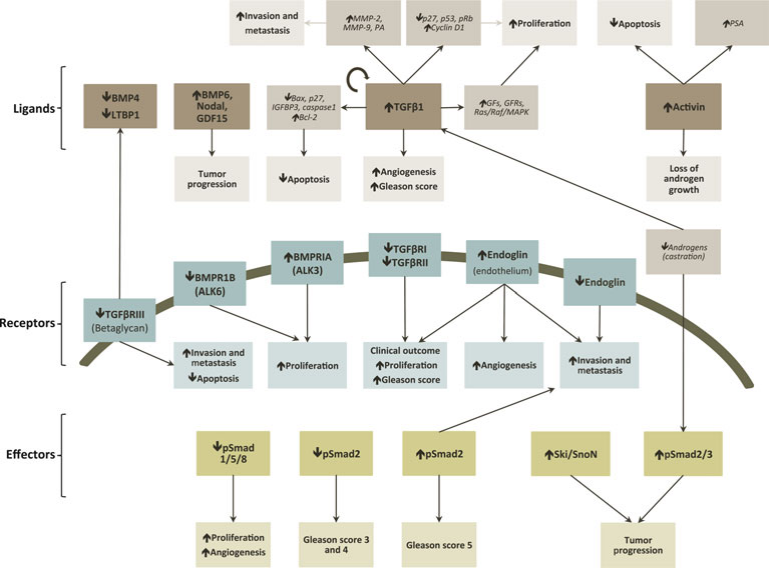
TGF-β signalling cascade alterations that lead to prostate cancer. Main effects or alterations related to the TGF-β signalling pathway that drive to prostate cancer hallmarks. Brown boxes: ligands; Blue boxes: receptors; Green boxes: downstream effectors of the TGF-β signalling pathway; Light brown boxes: alteration in other targets (italics). Grey, light blue and light green boxes: alteration in cancer cell hallmarks or effects related to the ligands/other targets, receptors or downstream effectors, respectively; Black arrows inside boxes: increase/decrease or activation/inactivation; Black arrows: effect or alteration. PSA, prostate-specific androgen; MMP-2, matrix metalloproteinase 2; MMP-9, matrix metalloproteinase 9; PA, plasminogen activator; pRb, protein retinoblastoma; BMP, bone morphogenetic protein; LTBP1, latent TGF-β binding protein 1; GDF15, growth differentiation factor 15; IGFBP3, insulin-like growth factor binding protein 3; GF, growth factors; GFR, growth factor receptor; MAPK, mitogen-activated protein kinase; BMPR, bone morphogenetic protein receptor; TGF-β R, TGF-β receptor; ALK, activin receptor-like kinase; EGFR, epidermal growth factor receptor.

In prostate cancer, there is a dramatic increase in TGF-β1 mRNA and protein levels, which are correlated with high Gleason score, bone metastasis, angiogenesis and poor clinical outcome 27,78 (Fig. 2). Exogenous TGF-β causes auto-induction in both normal and malignant cell lines at low concentrations. However, such effect is only seen at high concentrations in malignant cells 79. In prostate cancer, TGF-β-mediated apoptosis involves: (*i*) caspase 1 activation 80; (*ii*) up-regulation of pro-apoptotic factors (*e.g*. Bax, p27^KIP1^); and/or (*iii*) down-regulation of antiapoptotic factors (*e.g*. Bcl-2 and Bcl-xl) 51,81 (Fig. 2). Bcl-2 overexpression plays a role in the development of androgen-independent prostate cancer cells, conferring resistance to apoptosis by an antagonistic effect in caspase 1 activation, providing resistance to radio and chemotherapies 82. Moreover, loss of p27^KIP1^ in prostate cancer has been firmly established and regarded as a prognostic marker of increased recurrence and reduced survival 83. Even though most studies are centred in TGF-β, other ligands of the TGF-β superfamily may also play pivotal roles in prostate cancer (*e.g*. Activins, BMP6, GDF15 and Nodal/BMP16) 52,84.

### Receptors

Up to 30% of prostate cancer cases have down-regulation or absence of a TGF-β receptor, while no alterations in Smads are usually found 27. Mutations of TGF-βRII are common in lung and laryngeal cancers, but not in prostate cancer 52. Nevertheless, some prostate cancer cells express a truncated TGF-βRI mRNA transcript 85, lack a TGF-βRII gene 86, have TGF-βRs epigenetically regulated 87,88, or carry some sort of TGF-βRII mutation 89. The fact that TGF-βRI and TGF-βRII are decreased in metastasis *versus* primary tumours may indicate an active role of this alteration in cancer progression 90 (Fig. 2). Therefore, the absence of TGF-βRs in prostate tumour cells leads to growth inhibition resistance, thus resulting in clonal expansion of these cells 74,91–94. Acquired resistance can also be developed as a result of alterations in downstream genes such as p27 (repression), Cyclin D1 (induction), p53 and protein retinoblastoma (mutations) or *via* alterations in other pathways, like Akt/mTOR 83,95–97.

The BMPRs appear to elicit different responses, with a shift from BMPR1B towards BMPRIA being associated with cancer 98,99.

Several studies have also reported the loss of TGF-βRIII as the most common alteration during prostate cancer progression, being this alteration even more evident in metastasis 100 (Fig. 2). This loss occurs at both the mRNA and the protein levels and is subject of either direct or indirect epigenetic regulation. Knockdown of TGF-βRIII in prostate epithelial cells led to alterations in 101 genes associated with prostate cancer and its restoration decreased tumour growth, angiogenesis and increased apoptosis 100. Moreover, loss of TGF-βRIII correlates with disease state, metastatic disease and prostate-specific antigen (PSA) recurrence 100.

In a similar manner, endoglin levels are lower in prostate cancer cells *versus* normal prostate cells, and even lower in metastasis (Fig. 2). Endoglin has been found to inhibit invasiveness, metastasis formation and motility while increasing cell adhesion, neovascularization and growth 101–104. In contrast, levels are higher in endothelial cells, being this associated with ongoing angiogenesis 98,102.

### Effectors

Smad alterations in prostate cancer are also found, although not as extensively described as alterations in TGF-β ligand and its receptors. In the initial stages of prostate tumour development, ALK2-Smad1/5/8 signalling is promoted to increase the growth and neovascularization, whereas in late-stage tumours, there is a shift towards ALK5-Smad2/3 signalling that leads to the acquisition of malignant capabilities, namely enhanced invasiveness, migration and metastasis formation 101,103. Also, endoglin may play a pivotal role in modulating this equilibrium 102. Smad4 promoter methylation has also been reported 105. High levels of Ski, a co-repressor of Smad2/3 (preferentially Smad3), were detected only in prostate cancer cells 84 (Fig. 2).

### Interconnections between TGF-β signalling and other signalling pathways

The relationship between TGF-β and androgens is also relevant. For instance, TGF-β has been linked to castration-induced apoptosis. After androgen withdrawal, TGF-β and TGF-βRs mRNA levels were up-regulated, at least transiently 74,78,106 (Fig. 2). Androgens have also been found to down-regulate Smads expression and activation. Dihydrotestosterone bounds to AR and this complex was able to bind to active Smad3, inhibiting the association of Smad3 with Smad binding element and, therefore, blocking the signal. Also, DHT leads to the inactivation of *Sp1* suppressing its binding to TGF-βRII promoter, decreasing TGF-βRII levels 52,107,108. It has been recognized that the AR status determines the sensitivity of prostate cancer cells to TGF-β-mediated apoptosis 109 and the ability to evade is of paramount importance in the development of cancer, especially in prostate 27.

## TGF-β pathway is regulated by protein phosphatases

Transforming growth factor beta signalling regulates numerous cellular responses. Thus, its activity is tightly controlled by reversible phosphorylation as well as other post-translational modifications, such as ubiquitination, SUMOylation and acetylation 110. The TGF-β pathway is activated by phosphorylation at the membrane receptors level and at the cytosolic Smad proteins, its primary effectors. In contrast, dephosphorylation of TGF-β signalling players provides a counterbalance mechanism that limits the duration and intensity of the signal, contributing to its termination. Despite the long-standing suspected influence of protein phosphatases in TGF-β signalling, concrete data only started to emerge recently 111.

### Protein phosphatases and TGF-β pathway

**Figure 3:**
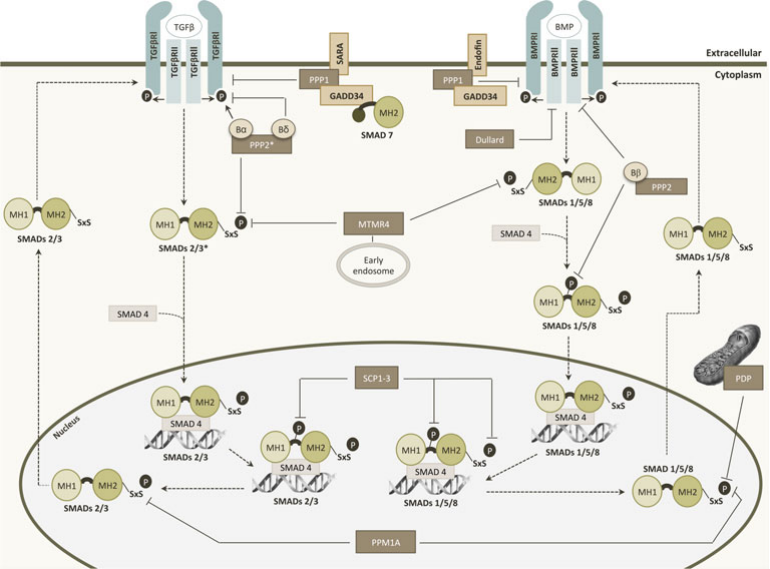
Regulation of TGF-β signalling by phosphatases. With exception of Dullard and PDP, which only act on BMP signalling, all phosphatases contribute to both pathways. PPP1 and Dullard act exclusively on receptors, whereas MTMR4, SCPs, PDP and PPM1A act exclusively on R-Smads. PPP2 can act both on receptors and R-Smads. *, PPP2 dephosphorylates the C-terminal SxS motif of Smad3 without interfering with Smad2 phosphorylation state; Dashed arrows: sequential events on TGF-β and BMP signalling pathways; Black arrows and black bars: phosphorylation or dephosphorylation events, respectively; Brown boxes: phosphatases involved; Beige boxes: PPP1 interacting proteins. PPP1, phosphoprotein phosphatase 1; PPP2, phosphoprotein phosphatase 2; PPM1A, protein phosphatase Mg^2+^/Mn^2+^ dependent 1A; PDP, pyruvate dehydrogenase phosphatase; MTMR4, myotubularin-related protein 4; SCP, small C-terminal domain phosphatase; TGF-βR, TGF-β receptor; BMPR, bone morphogenetic protein receptor; SARA, Smad anchor for receptor activation; GADD34, growth arrest and DNA damage-inducible protein 34.

An increasing number of protein phosphatases, particularly of the serine/threonine phosphatases family, have been reported to regulate the TGF-β pathway through interactions with both receptors and Smad proteins 112 (Fig. 3).

Phosphoprotein phosphatase 2 is a serine/threonine phosphatase in which the core structure consists of a catalytic and structural subunit. Although dimeric PPP2 can exist as such, it is normally associated with a third subunit that is regulatory. This subunit acts as a targeting and substrate-specifying unit, hence regulating this phosphatase and defining its physiological function. Phosphoprotein phosphatase 2 holoenzymes are important physiological regulators of proper checkpoint functioning during the cell cycle, which contributes significantly to their tumour suppressive abilities. It is well known that PPP2 inhibits both TGF-βRI and R-Smads 113 (Fig. 3). The regulatory B subunit family of PPP2 is composed by four homologous genes, two of which have opposite effects on TGF-β pathway. Bα (PPP2R2A) enhances TGF-β/Activin/Nodal signal by regulating the basal levels of ALK5, whereas Bδ (PPP2R2D) negatively modulates these signals by limiting the receptor activity 114. It has also been shown that TGF-βRI activation enables PPP2R2D binding to its cytoplasmic domain resulting in the phosphatase activation. Henceforth, PPP2R2D recruits PPP2C, PPP2A-β (PPP2R1B) and other regulatory subunits to bind and dephosphorylate p70^s6k^, a serine/threonine kinase crucial to progression of G_1_/S phases of cell cycle 115. Phosphoprotein phosphatase 2 also dephosphorylates the C-terminal SxS motif of Smad3 under hypoxic conditions without interfering with Smad2 phosphorylation state 113. In BMP signalling pathway, the regulatory subunit Bβ of PPP2 (PPP2R2B) has been recognized as an interactor of both BMPRII and Smad1 (Fig. 3). More specifically, it dephosphorylates BMPRII contributing to the inactive state of the receptor complex and reverses the phosphorylation by MAPK in the Smad1 linker region, enabling the Smad complex translocation into the nucleus 116.

Protein phosphatase Mg^2+^/Mn^2+^ dependent 1A (PPM1A) is induced by activated TGF-βRI (Fig. 3). This protein dephosphorylates the C-terminal SxS motif of activated Smads2/3 and Smads1/5/8 in the nucleus, thus promoting the dissociation of Smad heteromeric complexes. Subsequently, R-Smads undergo nuclear export, and the TGF-β-mediated anti-proliferative and translational effects are attenuated 117. Moreover, PPM1A dephosphorylates RanBP3, enhancing its aptitude to export nuclear Smad2/3 and promotes Smad1 proteasomal degradation 113,118. Pyruvate dehydrogenase phosphatase is a mitochondrial phosphatase that also acts on Smad1 C-terminal SxS motif (Fig. 3), being the molecular mechanism underlying this interaction still unclear 119. A member of dual specificity phosphatase family named myotubularin-related protein 4 (MTMR4) down-regulates the TGF-β signalling through interaction and dephosphorylation of activated Smads2/3 on early endosomes (Fig. 3). This dephosphorylation consequently blocks their nuclear translocation 120. Recently, it has also been described the MTMR4 involvement in the dephosphorylation of the SxS motif in BMP signalling R-Smads 121.

Also, a role for the aspartate-based phosphatases (FCPs and SCPs) in TGF-β signalling has been described (Fig. 3). In humans, there are three forms of small C-terminal domain phosphatases (SCP1-3) that bind with high affinity to Smad1, dephosphorylating it 118. Furthermore, they dephosphorylate linker region residues of R-Smads. More precisely, SCPs1-3 dephosphorylate the residues Ser245, Ser250 and Ser255 of Smad2 and analogues sites on Smad3, Thr8 and Thr179 of Smad3, and Ser187, Ser195, Ser206 and Ser214 of Smad1 122. Another aspartate phosphatase from the FCP family named Dullard interacts with the BMPRII resulting in its degradation and consequent inhibition of BMPRI signalling 123,124.

### PPP1 and TGF-β receptor activity

Past studies in *Drosophila melanogaster* Dpp (Decapentaplegic) signalling found that PPP1 catalytic subunit (PPP1C) acts as a negative regulator of TGF-β signalling through its binding to Smad anchor for receptor activation protein (SARA; Fig. 3). Also, disruption of this binding leads to the hyperphosphorylation of the Dpp type I receptor 125. Further studies in mammalian cells showed that SARA presents PPP1 to ALK5 receptor promoting its dephosphorylation and consequent signal attenuation. This targeting involves the inhibitory Smad7 and another PIP, GADD34 (PPP1R15B). It has also been shown that Smad7 recruits PPP1C to ALK1, inhibiting Smad 1/5/8 dependent pathway 126,127. The PIP that recruits PPP1 to ALK1 still needs to be elucidated; however, a Smad anchor for BMP signalling called Endofin was recently discovered. In a similar way of what happens to SARA, PPP1 also binds to Endofin and GADD34 to dephosphorylate the ALK3 and ALK6 receptors but without any intervention of the inhibitory Smad7 128.

This TGF-βRI dephosphorylation is a critical reversible mechanism in the regulation of TGF-β signalling in several cellular contexts such as cellular stress, DNA damage and cellular growth 112.

Interestingly, PPP1 (and also PPP2) activity is required to maintain endothelial cells in a resting state 129. Inhibition of PPP1 activity promotes endothelial cells migration consistent with its negative role in ALK1-induced activation of these cells 126.

## PPP1 and PIPs in prostate cancer

Protein dephosphorylation at the serine and threonine residues has important roles in regulating both cell survival and cell differentiation 130. Several evidence shows that PPP1 regulates multiple signalling pathways modulating cell apoptosis and differentiation 131. PPP1 regulates the two major human tumour suppressors, which disruption has also been associated with prostate cancer, p53 and pRb 132,133. Phosphoprotein phosphatase 1 dephosphorylates p53 to attenuate its transcriptional and pro-apoptotic activity 7. Also, pRb dephosphorylation by PPP1 negatively regulates apoptosis 6. Phosphoprotein phosphatase 1 dephosphorylates Akt, regulating its activity and also its downstream targets to suppress differentiation and promote apoptosis 134.

Alteration of PPP1 gene expression and activity is associated with multiple human cancers, including prostate cancer. Over the past two decades, it has become evident that PPP1 versatility is achieved by its ability to interact with multiple PIPs, many of which have been associated with crucial processes implicated in carcinogenesis, such as cell cycle, apoptosis and cell migration.

### NIPP1

**Figure 4:**
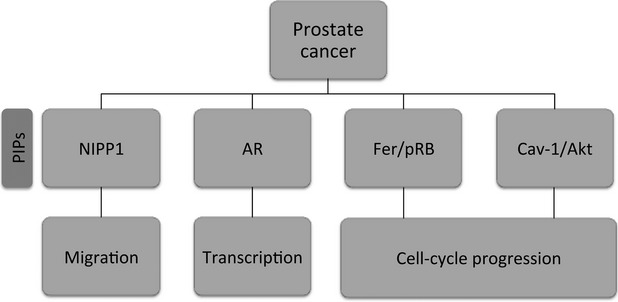
PPP1/PIPs complexes and their potential roles in prostate cancer. NIPP1, nuclear inhibitor of protein phosphatase 1; AR, androgen receptor; pRB, protein retinoblastoma; Cav-1, Caveolin-1.

Phosphoprotein phosphatase 1/Nuclear inhibitor of protein phosphatase 1 (NIPP1) complex has been recently described as a regulator of cell migration in prostate cancer cells 135. Nuclear inhibitor of protein phosphatase 1, is ubiquitously expressed and was initially characterized as a PPP1 inhibitor 136. Phosphoprotein phosphatase 1 has already been described as a regulator of cell polarity and migration, namely in controlling enteric nerve cell migration 137,138. Recently, Martin-Granados has described a role for PPP1/NIPP1 in directing migration of human cancer cells 135 (Fig. 4). Genetic disruption of PPP1 and NIPP1 decreases directional migration in PC3 cells. It has been shown that the PPP1/NIPP1 complex controls directed cell migration *via* up-regulation of Cdc42, and it has been suggested that the complex may contribute to the migratory properties of cancer cells 135.

### Androgen receptor

The AR is a ligand activated transcription factor, which plays a central role in prostate cancer development and progression, with androgen deprivation therapy being the standard treatment for prostate cancer 139,140. Chen *et al*. have shown that AR and PPP1 interact, and that PPP1 inhibition enhanced proteasome-mediated AR degradation. On the other hand, PPP1 overexpression increased AR expression and markedly enhanced AR transcriptional activity in prostate cancer cells 141 (Fig. 4). In addition, both AR and PPP1 undergo nuclear translocation when prostate cancer cells are stimulated by androgen. Phosphoprotein phosphatase 1 regulates AR protein stability and nuclear localization through its dephosphorylation at Ser650. Moreover, AR may function as a PPP1 regulatory subunit and mediate PPP1 recruitment to chromatin, where it can also modulate transcription and splicing events 141.

### Fer/pRb

Several lines of evidence implicate Fer, a tyrosine kinase, in the progression and growth of malignant tumours 142,143. Fer levels in malignant prostate tumours are significantly higher than those detected in benign tumours 17. Furthermore, down-regulation of Fer impaired the proliferation of prostate carcinoma cells and led to their arrest at the G0/G1 phase 17. Fer amino acid sequence analysis revealed two PPP1 binding motifs in the kinase domain, which most probably mediate the interaction of this kinase with PPP1 (Fig. 4). Another important protein, pRb, when hypophosphorylated sequesters E2F, a transcription factor required for G1-S transition, thereby preventing cell cycle progression in stress conditions such as ultraviolet radiation, ionizing radiation or hypoxia stress 73,144. Down-regulation of Fer potentiates the activation of PPP1 that has been shown to dephosphorylate and activate pRb leading to its growth suppressive state and cell cycle arrest in malignant cells 17,143,145. Therefore, the up-regulation of Fer in prostate cancer cells leads to the inactivation of PPP1 that culminates in pRb hyperphosphorylation resulting in a poor G1-S transition control and tumourigenesis 145.

### Cav-1/Akt

Caveolin-1 (Cav-1) is a ubiquitously expressed integral membrane protein, with antiapoptotic activity in prostate cancer cells, functioning downstream of androgenic stimulation 146,147. Caveolin-1 has been reported to be overexpressed in prostate cancer cells and is associated with disease progression 148,149. Li *et al*. found that Cav-1 interacts with and inhibits PPP1 and PPP2 150. Analysis of putative substrates for PPP1 and PPP2 revealed that Cav-1-mediated inhibition of PPP1 and PPP2 leads to an increase in PDK1, Akt and ERK1/2 activities. This unravels a novel mechanism of Akt activation in prostate cancer (Fig. 4). Through its binding and inhibition of PPP1 and PPP2, Cav-1 is able to maintain Akt activated, leading to sustained activation of downstream oncogenic Akt targets and increased cell survival 150. This mechanism could explain TGF-β-mediated apoptosis disruption by AKT pathway 151. An increase in Akt signalling has been correlated with poor clinical outcome in prostate cancer 110. These findings support the concept of PPP1 and/or PPP2 as tumour suppressor proteins and further support the notion that Cav-1 is an important metastasis-related gene.

## Conclusions

Prostate cancer is a common disease. In developed countries, it is the most incident cancer and the third in mortality. Up to 50% of elderly men have small clinically insignificant tumours and the lifetime risk of developing prostate cancer is, in the more developed areas, *circa* 7.8% 74,152. Most human prostatic carcinomas are initially responsive to androgen ablation therapy and radiotherapy. However, when prostatic carcinomas become castration-resistant, radical prostatectomy is the only option for treatment 153. Nonetheless, it has several side effects, including stricture of the vesico-urethral anastomosis, urinary incontinence and impotence 154. After metastasis formation no curative treatment is currently available being surgical or medical castration the current standard palliative treatment 74. Importantly, metastasis spread has been associated with most prostate cancer deaths 74,155. Recently, the U.S. Food and Drug Administration (FDA) approved a new therapeutic option to treat men with metastatic castration-resistant prostate cancer. However, this AR antagonist named Xtandi (MDV3100, enzalutamide, Astellas Pharma US, Inc., Northbrook, IL, USA) also has some side effects as seizures and weakness 156. Therefore, it is very important to identify non-androgenic and less aggressive therapeutic agents capable of reducing the proliferation of prostatic carcinoma 157. Currently, PSA levels, digital rectal examination and histological grading are the most commonly used detection techniques, although the utility of these methods (especially PSA) are currently in discussion, thus providing a need for new molecular biomarkers 21. A better knowledge of prostate cancer biology can provide the basis for such identification.

Among the different growth factors associated with normal prostate functioning, it has not been completely established which, if any, are associated with the acquisition of tumour cell autonomy. Transforming growth factor beta signalling pathway components have been regarded as possible targets for prostate cancer therapy for several years 158. In fact, genistein acts through activation of Smad1, thus suppressing prostate cancer cell invasion, in an ALK2-dependent way 102. Also, the delivery of oncolytic adenoviruses targeting TGF-β signalling resulted in less tumour burden, osteoclasts and trabecular and cortical destruction, thus representing a possible treatment for prostate cancer bone metastasis 159. The targeting of more general TGF-β effectors, like p53, has also been defended 160. The possibility of using TGF-β as a biomarker for prostate cancer has also been discussed for long 161. Serum levels correlate with tumour burden, metastasis and serum PSA. Also, TGF-β presence in semen is probably related with tumour stage 74.

Because of the critical effect of phosphatases and kinases on TGF-β pathway and cancer metabolic control, inhibitors of these molecules may represent good alternative treatment options. Although kinases continue to retain the primary focus as drug targets for cancer therapy, phosphatases are receiving increasing attention 162.

Despite all the efforts made by researchers and pharmaceutical companies in this area, the only FDA-approved drugs targeting a protein phosphatase are cyclosporine A and FK506. These drugs are used as immunosuppressors and inhibit PPP3 (calcineurin). Unfortunately, because of the numerous functions of PPP1 and PPP2 catalytic subunits, the long-term usage of non-selective or marginal selective enzyme inhibitors is associated with nephrotoxicity and hepatotoxicity because of the inhibition of a number of critical cellular processes. For these reasons, it will be interesting to target PIPs instead of protein phosphatases directly as they are more event, tissue and subcellular compartment specific 60. Nowadays, two targeted PPP1-PIP complexes have been described. The level of PPP1-GADD34 complex is diminished in cells treated with salubrinal, a small molecule that protect the cell from ER-stress-induced apoptosis 54. Studies have suggested that this compound constitutes a potential treatment for the herpes simplex virus infection as it inhibits the virus replication 54. The other complex involves PPP1 and histone deacetylases (HDACs) and is an attractive target to anti-tumour drugs. Trichostatin A, for example, disrupts the interaction between PPP1 and HDAC6 in glioblastoma and prostate cancer cells 54. Here, we presented increasing evidence that makes PPP1-PIP complexes attractive targets to pursue in the near future for prostate cancer therapy. However, PPP1-PIPs complexes previously described may not be suitable for targeting because of the several functions and the ubiquitous expression of those PIPs. Thereof, a deeper knowledge of the PPP1 interactome in both normal and malignant prostate is required. This information could lead to a new understanding of PPP1-PIPs complexes functions and which PIPs are better targets for more focused therapeutical approaches in prostate cancer.
